# Understanding factors that contribute to variations in bronchiolitis management in acute care settings: a qualitative study in Australia and New Zealand using the Theoretical Domains Framework

**DOI:** 10.1186/s12887-020-02092-y

**Published:** 2020-05-01

**Authors:** Libby Haskell, Emma J. Tavender, Catherine Wilson, Franz E. Babl, Ed Oakley, Nicolette Sheridan, Stuart R. Dalziel, On behalf of the Paediatric Research in Emergency Departments International Collaborative (PREDICT) nectwork, Australia

**Affiliations:** 1 Children’s Emergency Department, Starship Children’s Hospital, Private Bag 92024, Auckland, 1142 New Zealand; 2 University of Auckland, Auckland, New Zealand; 3 Murdoch Children’s Research Institute, Melbourne, Australia; 4 Department of Paediatrics, University of Melbourne, Melbourne, Australia; 5 The Royal Children’s Hospital, Melbourne, Australia; 6 College of Health, Massey University, Auckland, New Zealand; 7 Departments of Surgery and Paediatrics: Child and Youth Health, University of Auckland, Auckland, New Zealand

**Keywords:** Bronchiolitis, Acute care, Emergency department, Paediatric, Clinical guideline, Evidence-based practice, Theoretical domains framework

## Abstract

**Background:**

Bronchiolitis is the most common reason for infants under one year of age to be hospitalised. Despite management being well defined with high quality evidence of no efficacy for salbutamol, adrenaline, glucocorticoids, antibiotics or chest x-rays, substantial variation in practice occurs. Understanding factors that influence practice variation is vital in order to tailor knowledge translation interventions to improve practice. This study explores factors influencing the uptake of five evidence-based guideline recommendations using the Theoretical Domains Framework.

**Methods:**

Semi-structured interviews were undertaken with clinicians in emergency departments and paediatric inpatient areas across Australia and New Zealand exploring current practice, and factors that influence this, based on the Theoretical Domains Framework. Interview transcripts were coded using thematic content analysis.

**Results:**

Between July and October 2016, 20 clinicians (12 doctors, 8 nurses) were interviewed. Most clinicians believed chest x-rays were not indicated and caused radiation exposure (beliefs about consequences). However, in practice their decisions were influenced by concerns about misdiagnosis, severity of illness, lack of experience (knowledge) and confidence in managing infants with bronchiolitis (skills), and parental pressure influencing practice (social influences). Some senior clinicians believed trialling salbutamol might be of benefit for some infants (beliefs about consequences) but others strongly discounted this, believing salbutamol to be ineffective, with high quality evidence supporting this (knowledge). Most were concerned about antibiotic resistance and did not believe in antibiotic use in infants with bronchiolitis (beliefs about consequences) but experienced pressure from parents to prescribe (social influences). Glucocorticoid use was generally believed to be of no benefit (knowledge) with concerns surrounding frequency of use in primary care, and parental pressure (social influences). Nurse’s reinforced evidence-based management of bronchiolitis with junior clinicians (social/professional role and identity). Regular turnover of medical staff, a lack of ‘paediatric confident’ nurses and doctors, reduced senior medical coverage after hours, and time pressure in emergency departments were factors influencing practice (environmental context and resources).

**Conclusions:**

Factors influencing the management of infants with bronchiolitis in the acute care period were identified using the Theoretical Domains Framework. These factors will inform the development of tailored knowledge translation interventions.

## Background

Bronchiolitis is a common condition affecting infants less than 1 year of age, with presentations to small rural hospitals as well as large tertiary paediatric centres [1–3]. It is the most common reason for admission to hospital for infants aged less than 1 year. In New Zealand, there are > 70 admissions/1000 infants with bronchiolitis hospitalisation rates increasing year on year [4]. Māori (relative risk (RR) 3.0), Pacific (RR 4.3), and those infants living in the most deprived quintile (RR 4.7) are at most risk [5]. In Australia, bronchiolitis accounts for 56% of all admissions of infants aged less than 1 year [6]. Health care expenditure for bronchiolitis is predominantly confined to inpatient management of admitted patients, with estimates that in the United States alone this cost is US$1.7bn [7–9] accounting for approximately 100,000 infants admissions annually [8].

Management of bronchiolitis is well defined [7, 10, 11] and necessitates supportive care; respiratory support, supplemental hydration [12, 13] with medical and nursing involvement. Current international guidelines, [14–16] including a recent evidence-based guideline from Australia and New Zealand (Australasian Bronchiolitis Guideline) [17, 18] recommend against the use of inhaled salbutamol, inhaled adrenaline, oral glucocorticoids, antibiotics and chest x-rays (CXRs) as they are considered ineffective for routine care of infants admitted to hospital with bronchiolitis [12, 13, 17, 19–21]. Despite high quality evidence that these five therapies and management processes are ineffective and associated with harm, variation in practice continues. In Australia and New Zealand, data from over 3000 presentations to seven hospitals concluded that these five interventions were used at least once in 27 to 48% of bronchiolitis admissions [22, 23]. These data are consistent with international comparisons in North America, the United Kingdom and Europe [24] and highlight the gap between evidence-base and clinical practice.

There is little understanding of what works to improve clinical practice in paediatric emergency medicine (PEM) as evidence for effective dissemination and implementation methods in this setting is limited [25, 26]. What is clear from the knowledge translation (KT) literature is that interventions are more likely to be effective if theory is used in the development process. This allows the targeting of causal determinants of behaviour and facilitates an understanding of what works, for whom, and under what conditions, with resulting interventions then “tailored and theory informed” [27–29].

The Theoretical Domains Framework (TDF) [30, 31] has been designed to incorporate a wide range of theories relevant to behaviour change for use in implementation research. The TDF has a comprehensive structure of 14 theoretical domains from 33 behaviour change theories and 128 constructs. The framework has demonstrated strong explanatory and predictive powers across a number of healthcare settings and is particularly helpful when selecting interventions to improve practice change. This framework has been successfully trialled in the Australian ED environment to understand factors influencing the management of mild traumatic brain injury in adults and guide intervention development [32, 33]. A subsequent cluster randomised controlled trial of this intervention resulted in improvement in the uptake of practice recommendations [34]. The TDF has also been used to develop implementation interventions in the acute care setting of stroke management [35, 36]. For these reasons, this framework was considered appropriate to use to develop interventions aiming to reduce the evidence-practice gap in bronchiolitis management in Australia and New Zealand.

The aim of this study was to explore factors identified by ED and paediatric inpatient clinicians that may influence the uptake of five key evidence-based recommendations from the Australasian Bronchiolitis Guideline [17] (see Table 1). Secondary aims sought to identify key differences in influencing factors regarding location of hospitals (metropolitan, regional) and seniority (senior, junior) of clinicians (medical, nursing). Findings from this study will be used to develop a targeted, theory and evidence informed intervention aiming to reduce unnecessary use of therapies in the care of infants with bronchiolitis in Australian and New Zealand hospitals. Evaluation of the intervention will be assessed through a cluster randomised controlled trial, the protocol of which is reported separately [37].

**Table 1:** Key clinical recommendations from the Australasian Bronchiolitis Guideline

Clinical intervention	NHMRC strength of recommendation	GRADE quality of evidence	Guideline recommendation
**Salbutamol**	A	Strong	Do not administer salbutamol
**Antibiotics**	B	Conditional	Do not use antibiotics
**Glucocorticoids**	B	Strong	Do not administer systemic or local glucocorticoids (nebulised, oral, intramuscular or intravenous)
**Adrenaline**	B	Strong	Do not administer adrenaline (nebulised, intramuscular or intravenous)
**Chest x-ray**	D	Conditional	Chest x-ray is not routinely indicated

*NHMRC* National Health and Medical Research of Council

*GRADE* Grading of Recommendations, Assessment, Development and Evaluations

## Methods

### Study design

Qualitative study using in-depth, semi-structured interviews.

### Participants and sampling

Clinicians were eligible for inclusion if they were on an active practice roster working in either the ED or paediatric inpatient areas, in Australia and New Zealand as a doctor or nurse with responsibility for the medical and nursing management of infants with bronchiolitis. There were no exclusion criteria. We used a stratified purposeful sampling strategy from a range of metropolitan and regional hospitals who were part of the Paediatric Research in Emergency Departments International Collaborative (PREDICT) network, as well as sampling of junior and senior medical and nursing clinicians, in order to reduce selection bias. The objective was to recruit from four hospitals reflective of the range of hospitals where infants receive paediatric care across Australia and New Zealand; from two countries (Australia and New Zealand) and from two settings (metropolitan hospitals/tertiary provider of paediatric care with a paediatric ED and regional hospitals/secondary provider of paediatric care with a mixed adult/paediatric ED).

No incentives were provided to participants. Sampling continued until saturation of the data were reached, with the stopping criteria tested after the third and then each subsequent interview until there were three successive interviews with no new material identified [38].

### Recruitment

The clinical director of either the EDs or paediatric inpatient area was initially approached to confirm that their hospital would agree to take part in the study. An invitation letter was emailed to clinical directors of both ED and paediatric inpatient areas including a participant information sheet and consent form. The clinical directors, together with nursing leads, at each site forwarded study documentation to suitable medical and nursing staff for participation in the study. Interested individuals were invited to contact the research staff directly so that questions could be answered by phone or email. Participants opted into the study through completion of a consent form and gave verbal confirmation at the start of the interview.

Face-to-face interviews with clinicians were undertaken at an agreed time and location within their hospital. If face-to-face interviews were not possible clinicians were invited to be interviewed by telephone. Three researchers (LH, EJT, CW) conducted the interviews, with two researchers being present at each interview. All interviews were undertaken in English and were digitally recorded. LH is a paediatric emergency nurse practitioner with extensive experience of managing infants with bronchiolitis. EJT is a post-doctoral implementation researcher who has completed qualitative studies using clinician interviews such as this, and CW is a research coordinator with a nursing background.

### Interview content

The interview guide consisted of two parts (see Additional file 2: Interview schedule). First, broad questions were asked about bronchiolitis management and variability in practice. Second, clinicians’ perspectives were sought on why the five evidence-based bronchiolitis recommendations were being followed, or not. Their perceptions of the factors influencing their colleagues’ decision to order therapies and interventions were explored. The structure of the interview guide was informed by the TDF with questions formulated to explore domains. Practicing PEM clinicians (SRD, EO, FEB) and the research team reviewed and revised the interview schedule, which was subsequently piloted with a senior nurse and doctor not associated with the study. These interviews were not analysed.

### Data analysis

The interview recordings were de-identified and transcribed verbatim. Checked transcripts were uploaded into NVivo 11 qualitative data analysis Software (QSR International Pty Ltd., London, United Kingdom) to manage and facilitate the analysis.

Data were analysed using inductive thematic analysis and an iterative process. Two researchers (LH/EJT) independently reviewed the transcribed interview and open coded text relevant to each of the recommended practices and the factors influencing those practices. The TDF offered an analytic framework to code these factors. When text was relevant to more than one domain, it was cross indexed. Coding was discussed by interviewers after the first five interviews to ensure consistency. Discrepancies were identified, discussed and consensus was reached. Saliency analysis confirmed the degree to which each code occurred, and implied importance (frequently mentioned or deemed important by participants or researchers) [39]. Important factors within a domain were highlighted by verbatim quotations [40].

The Standards for Reporting Qualitative Research (SRQR) has been followed and checklist completed (see Additional file 1: SRQR checklist) [41].

Central ethics review and approval for the project was undertaken in Australia and New Zealand (see Declarations section for further details).

## Results

### Participants

Four hospitals were approached, all agreed to participate. Thematic saturation was reached at 20 interviews (see Table 2), conducted between July and October 2016. Face-to-face interviews were conducted with 17 clinicians and telephone interviews with three. The median interview duration was 30 min with a range of 20 to 49 min.

**Table 2:** Characteristics of participants

Hospital	Country	Level^**1**^	Area^2^	Local bronchiolitis guideline	Senior Medical Officer^3^	Resident Medical Officer	Senior registered nurse^4^	Registered nurse	Total
1	New Zealand	Metro	Paediatric ED	Yes	1	1	1		3
1	New Zealand	Metro	Paediatric inpatient ward	Yes	1			2	3
2	New Zealand	Regional	Mixed adult/paediatric ED	No^5^	1	1		1	3
2	New Zealand	Regional	Paediatric inpatient ward	No^5^	3			2	5
								New Zealand *n* = 14	
3	Australia	Metro	Paediatric ED	Yes	1			1	2
3	Australia	Metro	Paediatric inpatient ward	Yes	2		1		3
4	Australia	Regional	Mixed adult/paediatric ED	No^5^					0
4	Australia	Regional	Paediatric inpatient ward	No^5^	1				1
								Australia *n* = 6	
			**TOTAL**		**10**	**2**	**2**	**6**	**20**

^1^Level – Metropolitan/major city (metro) or Regional

^2^Area – Paediatric inpatient ward or Emergency Department (ED) (paediatric ED OR mixed adult and paediatric ED)

^3^Senior Medical Officer – Consultant Paediatrician or Emergency Physician (> 10 years post graduate experience)

^4^Senior registered nurse – nurse working in senior nursing position (> 10 years post graduate experience)

^5^Use local tertiary hospital’s guideline

Each of the recommended practices from the Australasian Bronchiolitis Guideline had its own patterns of influencing factors. Additional file 3: Tables S1 to S5 lists the factors perceived to influence practices, arranged by TDF domains and clinician group. Explanatory quotations have been included to further describe clinician’s beliefs. Minor changes to quotations have been made to ensure readability with the clinicians intended statement remaining unchanged and in italics. The following paragraphs summarize our findings. Responses were consistent across both countries.

#### Chest x-ray use in infants with bronchiolitis

The majority of clinicians did not see routine CXR as indicated for infants with bronchiolitis.

### Factors influencing practice

The key factors perceived to influence practice of performing a CXR in infants with bronchiolitis were predominantly grouped within five domains: beliefs about consequences, knowledge, skills, social influences, and environmental context and resources (see Additional file 3: Table S1).

There were widely held concerns about the known risks of radiation (beliefs about consequences).

*What concerns you is if the child has been in eight times for bronchiolitis and they’ve had six x-rays, and you, kind of, think that’s getting unnecessary.* (Senior Medical Officer (SMO), Paediatric Inpatients, Regional)

There were some who believed that as a CXR involved only a small amount of radiation, it was not a problem (beliefs about consequences).

*They’re just doing extra things that are unnecessary and costly and maybe just have a little bit of morbidity in the sense you’ve given them a small amount of radiation.* (SMO, Paediatric Inpatients, Metropolitan)

The majority of nurses and doctors expressed concerns about missing an alternative diagnosis or, if an infant was in significant respiratory distress, a CXR could be justified and might assist in confirming the diagnosis (beliefs about consequences).

*I think they’re worried about deterioration. And I think worried about whether a child should be on IV antibiotics.* (SMO, Paediatric ED, Metropolitan)

Both doctors and nurses stated that CXRs were more likely to be undertaken by clinicians who had a lack of knowledge and experience in caring for infants with bronchiolitis (knowledge).

*Yeah, I think it’s lack of knowledge and seniority, really. The more junior you are, you, I guess, tend to do more things, because you think it’s the right thing to do and it might not be the right thing to do.* (Senior Registered Nurse, Paediatric ED, Metropolitan)

Several senior nurses and doctors perceived there was often a lack of competence and confidence in caring for infants with bronchiolitis which led to the increased use of CXRs (skills).

Several clinicians considered that parental pressure was a factor in CXRs being performed (social influences). Either they were expecting a CXR (e.g. had been advised by their primary health care provider that one would be completed in the ED) or that parents felt reassured if a CXR was completed.

*So there are parents that come in with expectations.* (Resident Medical Officer (SMO), Paediatric Inpatients, Metropolitan)

Several environmental context and resource factors were identified that were thought to influence a clinician’s decision to order a CXR. In both metropolitan and regional hospitals, time pressure to make decisions in ED (all sites have government imposed ED length of stay targets of between 4 and 6 h), reduced after hours support for junior medical staff, and turnover and rotations of staff were thought to contribute to infants receiving a CXR.

*A brand-new Senior House Officer who has only seen a handful of bronchiolitis, who’s on overnight, who’s got a baby who’s working [hard] and has a fever, then I think that they will be more inclined to do an x-ray.* (SMO, Paediatric Inpatients, Regional)

Specific influencing factors for regional hospitals were identified with more doctors being trained overseas where practices differ, staff who were not paediatric trained, and the challenge of distance to tertiary care providers.

*I suppose from a retrieval’s perspective, you really want to make sure it’s bronch and nothing else. And you’re not going to get a surprise on route as well.* (SMO, Paediatric Inpatients, Metropolitan)

#### Salbutamol use in infants with bronchiolitis

There was variability in the self-reported use of salbutamol. Some clinicians stated they would not give it to an infant with bronchiolitis, while others stated they would trial it on some. Senior and junior doctors shared both perspectives.

### Factors influencing practice

The key factors thought to influence clinicians’ decisions on whether to use salbutamol in infants with bronchiolitis centred on four key domains: beliefs about consequences, knowledge, social/professional role and identity, and social influences (see Additional file 3: Table S2).

Senior doctors showed variation in their reported use of salbutamol. Some saw benefits in using salbutamol in infants with a history of atopy or those closer to 1 year of age (beliefs about consequences). From personal experience, they felt it was worth trying if it enabled an infant to be discharged home rather than be hospitalised.

*If someone can be made better and go home, then it’s worth trying.* (SMO, Paediatric ED, Metropolitan)

Some senior doctors indicated that having a new guideline would not change their own practice in the use of salbutamol and there was no harm in trialling it.

Others believed an evidence-based guideline stating not to use salbutamol would be of benefit to guide practice for both junior and senior clinicians in metropolitan and regional hospitals (knowledge). Additionally, being aware of normal illness progression, and knowledge that salbutamol is not effective, would guide practice decisions.

*Because most of us don’t at a more senior level [give salbutamol], if we see a deteriorating bronchiolitis, we’re more willing to accept that as progression of the underlying condition, as opposed to lack of treatment with salbutamol.* (SMO, Paediatric Inpatients, Metropolitan)

Nurses and doctors with a wide range of experience levels perceived a lack of knowledge in regard to the evidence for salbutamol use in infants with bronchiolitis.

*If you are going to try a bronchodilator, why are you doing this? And what’s the evidence behind it and understanding the lack of evidence behind it, and doing that in conjunction with a senior clinician.* (SMO, mixed adult/paediatric ED, Regional)

Some nurses felt that knowledge was a factor when junior doctors treated infants with bronchiolitis in the same way they would an older child with viral induced wheeze or asthma.

*I think they are, kind of mixing the asthma/viral induced wheeze with the bronchiolitis one; so we were going by our experience with the patient whereas I feel like sometimes they are drawing on experience with something else.* (Registered Nurse (RN), Paediatric Inpatients, Metropolitan)

Senior nurses and doctors discussed the crucial role that nurses have in guiding junior doctors’ practice and in questioning their clinical decisions and treatments (social/professional role and identity).

*And in fact, our nurses are one of the most influential group. Because they question the juniors; the junior doctors.* (SMO, Paediatric ED, Metropolitan)

There were mixed views from nurses in regard to questioning doctors’ orders for salbutamol. Most senior nurses felt comfortable but there were times junior nurses did not feel listened to.

*There are so many children who are charted salbutamol where the nursing staff are saying, “Look, this child isn’t responsive” but then one of the medical team will go and hear something completely different from what the nurses hear, so they’ll say, “Yes, they are partially responsive. You should continue.” So, nursing staff will continue, but still not think it’s effective.* (RN, Paediatric Inpatients, Metropolitan)

Nurses and doctors discussed pressure from both parents and clinicians to trial salbutamol (social influences). Parents had sometimes been given salbutamol for a previous episode of bronchiolitis, or in an older child with wheeze, and believed it made a positive difference. Pressure on clinicians came from wanting to do something to help an infant, or pressure from other clinicians questioning whether they had tried salbutamol.

*I think the commonest reason for its use, well, the two things is people wanting to give them something, so parental pressure and clinicians’ internal pressure to give some kind of treatment for an illness that there is actually no additional treatment for and misunderstanding about the idea that it’s not actually salbutamol responsive wheeze.* (SMO, mixed adult/paediatric ED, Regional)

#### Antibiotic use in infants with bronchiolitis

The majority of clinicians stated they did not prescribe antibiotics for infants with bronchiolitis, but noted they saw many infants being given antibiotics by other clinicians.

### Factors influencing practice

The key factors perceived to influence antibiotic use in infants with bronchiolitis were grouped in three domains: beliefs about consequences, social influences, and knowledge (see Additional file 3: Table S3).

Amongst nurses and doctors there was a consistent belief that there is no benefit in administering antibiotics to infants with bronchiolitis. Most clinicians were comfortable giving advice to stop a course that had been started. There were mixed beliefs about the consequences of antibiotic use; the majority of clinicians felt strongly about reducing antibiotic use due to increasing concerns about antibiotic resistance, although some did not share this concern (beliefs about consequences).

*48 hours of antibiotics in terms of the big picture of antibiotic resistance isn’t going to make that much of a difference.* (SMO, Paediatric ED, Metropolitan)

One senior doctor spoke of high antibiotic prescribing for infants with bronchiolitis in his region, which he believed was due to a high bronchiectasis rate in the local population.

*We do have quite a high antibiotic prescribing rate but we think that we’ve also got a high bronchiectasis problem. So being too strict about it being just viral, I might leave our high Māori deprivation, smoke exposed kids to not being appropriately treated for early bronchiectasis.* (SMO, Paediatric Inpatients, Regional)

Doctors and nurses discussed the pressure they experienced from parents who wanted antibiotics to be prescribed, or if a course had started, to continue (social influences).

*I think they do [in regard to doctors having a concern about the consequences of giving antibiotics] but they are focusing on the short term as opposed to the long term, so in the short term they’re keeping the family happy.* (RN, Paediatric Inpatients, Metropolitan)

Some clinicians perceived a lack of knowledge and limited experience in the management of bronchiolitis could result in a CXR being performed and antibiotics subsequently being prescribed (knowledge).

*Which is why so many children get treated with antibiotics, I think. There’s concern that there’s not quite enough wheeze. That leads to an x-ray. There’s abnormalities on the x-ray. That leads to treatment with antibiotics.* (Senior RN, Paediatric Inpatients, Metropolitan)

#### Glucocorticoid use in infants with bronchiolitis

The vast majority of clinicians reported that they did not use glucocorticoids for infants with bronchiolitis, but did see many infants who had been prescribed this by other clinicians.

### Factors influencing practice

The key factors identified that influenced clinicians in regard to glucocorticoid use were grouped within six domains: knowledge, beliefs about consequences, beliefs about capabilities, social/professional role and identity, environmental context and resources, and social influences (see Additional file 3: Table S4).

There was a widely held belief among clinicians that there was no benefit to be gained from the use of glucocorticoids in infants with bronchiolitis. There was also a concern about the possible consequences of glucocorticoids use (knowledge, beliefs about consequences).

*So on a ‘primum non nocere’ principle that steroids could actually predispose to immune suppression, I saw one fatal pneumonia from recurrent courses of Redipred prescribed to a baby that had barely reached term, or not long past it.* (SMO, Paediatric Inpatients, Regional)

Several clinicians discussed the use of glucocorticoid in the community and expressed concerns around repeated use.

*I was very surprised by the high use of it in the community, you know, often the families were coming and not only having had steroid but have a stash of steroid at home and are using that.* (SMO, Paediatric Inpatients, Regional)

Because doctors believed there was no need to prescribe glucocorticoids, they were comfortable in advising a family to stop a course that had been started (belief about capabilities). They were conscious of the need to discuss this in a manner that preserved the existing relationship with a family’s primary healthcare provider (social/professional role and identity).

*You need families to trust their General Practitioners. So you don’t want to run them down and say, “This is ridiculous, stop it”.* (RMO, Paediatric ED, Metropolitan)

One clinician discussed how to approach stopping glucocorticoids with a family, and the importance of listening and acknowledging the family’s perspective.

*I always sort of think, would you give this to your own kids, would I take this myself?* (SMO, Paediatric Inpatients, Regional)

Environmental context was an important factor for some regional hospitals that had staff who had trained and worked in countries where beliefs and practices in the use of glucocorticoids differed (environmental context and resources).

*We’ve had quite a few American consultants come through who have quite different opinions, so it’s actually quite difficult for our juniors.* (SMO, mixed adult/paediatric ED, Regional)

Several clinicians perceived there was pressure from families to prescribe glucocorticoids (social influences) and that this was often based on a family’s past experience.

*Especially if you’ve got a kid with recurrent bronchiolitis the parents will go “This is what works. This is the only thing that’s worked in the past. We’ve tried this and this; we always have to go to steroids at the end of the day”.* (RN, mixed adult/paediatric ED, Regional)

#### Adrenaline use in infants with bronchiolitis

There was very limited discussion around the use of adrenaline in uncomplicated bronchiolitis due to this therapy being rarely used in Australian and New Zealand hospitals except in peri-arrest of a critically unwell infant.

#### General comments around caring for an infant with bronchiolitis

Interviewers attempted to direct the interviews to the five recommendations from the Australasian Bronchiolitis Guideline as detailed previously (see Table 1) [17]. Throughout the interviews, many participants spoke about more general issues and the challenges of caring for infants with bronchiolitis. Given the frequency of these views, they were considered important, and coded.

The key factors that influenced the overall care of infants with bronchiolitis were: beliefs about consequences, knowledge, social/professional role and identity, environmental context and resources, and skills. (see Additional file 3: Table S5).

There was a concern from clinicians that the infant would deteriorate and they should be intervening (belief about consequences).

*This kid is looking quite sick, I should be doing something.* (SMO, Paediatric ED, Metropolitan)

There were very positive responses to the development of an Australasian Bronchiolitis Guideline [17] with doctors and nurses from ED, inpatient paediatrics, metropolitan and regional hospitals believing that this would improve the care of infants with bronchiolitis. Clinicians said the guideline would increase overall consistency of care across states and countries (knowledge).

*Once you have very clear protocol type guideline as to what to do and when to do it, then it gives people a recipe to follow and they’re more likely to follow it.* (SMO, Paediatric Inpatients, Metropolitan)

Nurses reported that a guideline would provide sound evidence to support their discussions with doctors and inform management.

Key differences in the management of bronchiolitis due to location were discussed. Emergency clinicians spoke of the challenges in mixed adult/paediatric EDs and of feeling less confident in the care of infants, relative to adults (knowledge).

Concerns related to the recognition of illness, and severity of illness, and undertaking procedures, such as inserting a nasogastric tube in an infant. Doctors and nurses highlighted the importance of positive relationships between clinicians (social/professional role and identity) and a willingness to seek advice.

*It’s probably a bit of lack of knowledge and lack of experience; you’ve not seen enough, you’re just worried. They can look quite bad and you are in a position where you don’t really want to miss something, although you’ve really got nothing to miss.* (RMO, Paediatric ED, Metropolitan)

Regional hospitals highlighted the challenges of distance and time in transferring critically unwell infants, and suggested this might influence care. For example, a CXR undertaken as a routine investigation prior to transfer, which commonly occurs (environmental context and resources).

*It’s terrifying! (in response to “it must be quite stressful at night?”)*. (RMO, Mixed ED, Regional)

Additionally, regional hospitals were more likely to have locum or overseas trained staff with frequent rotations, creating challenges in maintaining staff competencies in line with best practice guidelines.

Role modelling by senior clinicians was highlighted as a way of developing skills (skills); listening to how other clinicians interacted with families and emulating this in their own practice was described as helpful.

*But I think we also under describe what we are doing. Like I think honouring nursing care at its best, we do that insufficiently at times. So for example, I think it should be phrased quite positively. We are maintaining fluids and all those regimens.* (SMO, Paediatric Inpatients, Regional)

Many clinicians also discussed the increased use of high-flow oxygen therapy and its place in the care of infants with bronchiolitis.

*Machines that go beep, and it looks impressive to the family, and we have a real perception that it's one of those.* (SMO, Paediatric Inpatients, Regional)

## Discussion

This study used the TDF to explore factors, real or perceived, that influence the management of infants with bronchiolitis in relation to CXR, salbutamol, antibiotics, glucocorticoids and adrenaline. Despite wide variation in clinical practice, we are not aware of any other study that has investigated this issue. Five domains were identified as consistently important in four of the five interventions known to be of no benefit in the care of an infant with bronchiolitis: beliefs about consequences, knowledge, social/professional role and identity, environmental context and resources, and skills. Using the TDF, our study has generated knowledge and understanding of what influences bronchiolitis management in Australian and New Zealand settings, and provides an ideal platform to design interventions targeted to improve practice. As practice variation occurs in other countries, such as the United Kingdom, Canada and the United States, while needing confirmation, these findings are likely to have high relevance.

The domain of beliefs about consequences was consistently found to be important, particularly in relation to the use of CXR. Clinicians face a challenging task when an infant with bronchiolitis presents in the acute care setting – they are generally unknown to them, time pressure is a feature of the ED environment, and the clinician is concerned about missing a more serious diagnosis. Major influencing factors are likely to be fear of missing a pneumonia as well as the relative luxury of being able to obtain a CXR in the ED [42]. Our interviews highlighted the belief that the frequency with which CXRs are performed was primarily due to concern about missing a more serious diagnosis, and believing a CXR would further assist in diagnosis. The unnecessary use of CXR exposes infants to radiation, increases financial cost, increases the time of medical care [43] and increases unnecessary use of antibiotics [44]. Doctors and nurses of all levels of experience commonly expressed concerns about radiation exposure and inappropriate antibiotic use. Implementation of an Australasian evidence-based bronchiolitis guideline provides the ideal platform to improve clinicians’ knowledge of bronchiolitis leading to confidence in diagnosis, reduced fear of missing an alternative diagnosis such as pneumonia, and improved bronchiolitis management.

It was reassuring that the majority of clinicians felt comfortable to give advice to stop a course of antibiotics, and were aware of the importance of managing this in a way that maintained the existing relationship between the family and primary care provider.

The second most common domain was around knowledge of bronchiolitis and its management. One of the factors contributing to this may be that ED clinicians deal with a broad spectrum of conditions, which pose challenges in keeping up-to-date with current practice. This was highlighted in a recent Australian study investigating the evidence-based management of minor traumatic brain injury in EDs [32]. From our interviews, there was a belief that some doctors and nurses lacked confidence in managing sick infants in mixed adult/paediatric EDs where the majority of patients presenting are adults. This was an issue raised by both doctors and nurses. Another issue related to junior doctors, was their frequent rotations through specialties, and the importance of knowledge development in the care of infants, including those with bronchiolitis. Overall, there was a common belief that all staff needed to be up-to-date with current clinical guidelines with acknowledgement of the challenge in achieving this. The consistently affirmative opinion of clinical guidelines from both nurses and doctors is expected to positively influence the successful implementation of the Australasian Bronchiolitis Guideline aiming to improve the management of infants with bronchiolitis.

Out of the five interventions known to be of no benefit, salbutamol provided the most variation in clinicians’ responses. The belief that salbutamol was effective and worth trialling on some infants was firmly held by some senior doctors (ED and inpatient paediatrics; metropolitan and regional) who stated that a guideline would not change their practice. In contrast, other senior doctors felt equally as strongly about not administering salbutamol, and welcomed a guideline that supported their belief that salbutamol was not effective and shouldn’t be used.

Social/professional role and identity and the importance of collaborative care and clinician relationships was also an important feature in the care of infants with bronchiolitis. Nurses said they felt valued when asked for advice on bronchiolitis management by their medical colleagues, but some also expressed concern that their clinical assessments were sometimes ignored, in particular in relation to the ineffectiveness of salbutamol. At times junior doctors said they felt torn between practicing evidence-based medicine, and having a senior colleague advise them to the contrary. This mainly occurred in regard to the use of salbutamol and CXR.

The social influences domain was evident in findings from four clinical therapies: CXR, salbutamol, antibiotics and glucocorticoids. Parental or care giver pressure to “do something” when advocating for their sick infant by either wanting medication or a CXR was highlighted. Clinicians listened to the requests of families and at times felt challenged by these interactions. Despite knowing specific interventions were not advised, they felt helpless just offering supportive care. Some junior doctors felt strategies for discussing bronchiolitis with a family would be helpful, with the focus being on the value of supportive care, and the importance of minimising unnecessary interventions. All hospitals used either their own local bronchiolitis family information sheet or one from another hospital. Clinicians felt these to be of value in reinforcing information given verbally, and helping to reduce parent and caregiver stress.

A recent study conducted in Wales using multi-faceted education (developed by the study authors) to reduce investigations (CXR, naso-pharyngeal aspirate, bloods, blood gas and urine) in infants with bronchiolitis, reported a 21% decrease in their use nationally. Directly approaching clinicians for feedback to improve the bundle, as well as increasing involvement with the multi-disciplinary team (doctors and nurses) was recommended [45]. Additionally, Mittal et al reports a reduction in CXR, bronchodilator, glucocorticoid and antibiotic use with the introduction of a clinical practice guideline and multi-faceted education in the United States (designed by a multi-disciplinary team from key stakeholders of a Bronchiolitis Task Force) [46]. We suggest that by specifically targeting those factors which all clinicians believe lead to investigations and therapies being undertaken could lead to further improvement.

The findings from this qualitative study will be used to guide the development of a tailored, theory informed KT intervention aiming to reduce the use of the five evidence-based therapies known to be of no benefit in the management of infants with bronchiolitis. Using the TDF to explore factors influencing the uptake of evidence-based care of adults with minor traumatic brain injury in the ED setting, then developing intervention components to address these factors has previously been undertaken in Australia [33]. We will use this approach for the design of interventions to promote evidence-based practice in bronchiolitis care, which will be evaluated in a cluster randomised controlled trial in both the ED and paediatric inpatient setting in Australia and New Zealand [37].

Although this study has clear strengths such as using a theoretical framework to explore factors believed to influence practice as well as sound interview techniques and rigorous coding to check interrater correlation, there are inevitably potential limitations. Findings report only the perceptions of those clinicians who participated. However, we are reassured that saturation was reached after 20 interviews, especially as clinicians had a wide variety of experience, practice type and location, and, suggest their views are likely to reflect the wider group of clinicians managing bronchiolitis in Australia and New Zealand. Due to the challenges of conducting interviews in two countries, there were slightly fewer Australian participants. We are aware that factors influencing practice can change over time. The interviews were completed during the period when the Australasian Bronchiolitis Guideline [17] was being developed and the effect that this may have had on practice across Australia and New Zealand EDs and paediatric inpatient areas cannot be underestimated.

## Conclusion

Using the TDF, factors thought to influence the management of infants with bronchiolitis in both ED and paediatric inpatient areas have been identified. Each of the interventions has its own pattern of influence but demonstrates similarities across all with key factors relating to beliefs about consequences, knowledge, social/professional role and identity, social influences and skills. Identification of these factors provides theoretically-derived goals for the development of a KT intervention to address the issues and measure effect and influence on practice**.**

## Supplementary information

**Additional file 1.** Standards for Reporting Qualitative Research checklist.

**Additional file 2.** Interview schedule.

**Additional file 3 Table S1.** Factors thought to influence practice in caring for infants with bronchiolitis in regard to chest x-ray. **Table S2**. Factors thought to influence practice in caring for infants with bronchiolitis in regard to salbutamol use. **Table S3.** Factors thought to influence practice in caring for infants with bronchiolitis in regard to antibiotic use. **Table S4**. Factors thought to influence practice in caring for infants with bronchiolitis in regard to glucocorticoid use. **Table S5.** Factors thought to influence practice in caring for infants with bronchiolitis in general.

## Data Availability

The datasets generated and/or analysed during the current study are not publicly available due to persons being potentially identifiable. Summary data available from the corresponding author on reasonable request.

## Abbreviations

CXR: Chest x-ray
ED: Emergency department
KT: Knowledge translation
RMO: Resident medical officer
RN: Registered nurse
SMO: Senior medical officer
TDF: Theoretical domains framework
