# Supplementary material for: Understanding factors that contribute to variations in bronchiolitis management in acute care settings: a qualitative study in Australia and New Zealand using the Theoretical Domains Framework
Source: BMC Pediatr. 2020 May 1;20:189. doi: 10.1186/s12887-020-02092-y (PMC7193400; doi:10.1186/s12887-020-02092-y)
Supplement: Supplementary file 1 — Additional file 1. Standards for Reporting Qualitative Research checklist. [file 12887_2020_2092_MOESM1_ESM.docx]

| Additional file 1: Standards for Reporting Qualitative Research checklist |  |
| --- | --- |
| <http://www.equator-network.org/reporting-guidelines/srqr/> |  |
|  |  |

**Title and abstract Page No(s)**

| **Title** | 1 |
| --- | --- |
| **Abstract** | 3-4 |

**Introduction**

| **Problem formulation** | 5-6 |
| --- | --- |
| **Purpose or research question** | 7 |

**Methods**

| **Qualitative approach and research paradigm** | 7 |
| --- | --- |
| **Researcher characteristics and reflexivity** | 10 |
| **Context** | 9 |
| **Sampling strategy** | 9-10 |
| **Ethical issues pertaining to human subjects** | 9, 35 |
| **Data collection methods** | 10, 11 |
| **Data collection instruments and technologies** | 10, 11 |
| **Units of study** | 12, 13 |
| **Data processing** | 10, 11 |
| **Data analysis** | 11 |
| **Techniques to enhance trustworthiness** | 11 |

**Results**

| **Synthesis and interpretation** | 14-28 |
| --- | --- |
| **Links to empirical data** | 14-28  Additional file 2.S1-S5 |

**Discussion**

| **Integration with prior work, implications, transferability, and contribution(s) to the field** | 29-33 |
| --- | --- |
| **Limitations** | 32-33 |

**Other**

| **Conflicts of interest** | 35 |
| --- | --- |
| **Funding** | 35 |
