# Supplementary material for: Understanding factors that contribute to variations in bronchiolitis management in acute care settings: a qualitative study in Australia and New Zealand using the Theoretical Domains Framework
Source: BMC Pediatr. 2020 May 1;20:189. doi: 10.1186/s12887-020-02092-y (PMC7193400; doi:10.1186/s12887-020-02092-y)
Supplement: Supplementary file 2 — Additional file 2. Interview schedule. [file 12887_2020_2092_MOESM2_ESM.docx]

**Additional file 2: Interview schedule**

**Interview schedule for Emergency Department (ED) and General Paediatric inpatient nursing and medical staff

Introduction**

Thank you for agreeing to be interviewed for this study. As outlined in the explanatory statement, this study aims to identify and explore the factors influencing the management of infants with bronchiolitis.

You have signed a consent form previously. Can you confirm that you are still happy to proceed with the interview?

You do not have to answer every question and can cease the interview at any time. If you need to attend to an urgent matter, we can stop the interview and recommence it later.

We will talk to you about how infants with bronchiolitis are managed in your setting and the factors that influence their management. We are interviewing nursing and medical staff to learn from their experiences and are including a wide range of hospitals to get a broad view.

Before I start do you have any questions you would like to ask me?

**Demographic / Background Information**

For the purpose of this interview we are talking about children < 1 year of age. Do you care for infants with bronchiolitis?

**Management of infants with bronchiolitis – open questions**Can you talk me through how infants with bronchiolitis are managed in your ED / inpatient area?

Which health professionals are involved in the management of these infants and, briefly, what do they each do?

For instance, what happens when a patient with bronchiolitis presents to the ED department / is admitted to the inpatient area?

*Who is responsible for assessing the infant and making a plan for care?*

*What happens once they are assessed, what happens next?*

What do you think are the key actions/decisions when managing an infant with bronchiolitis?

Could we now discuss some of the different stages of managing an infant with bronchiolitis in more detail?

| Guideline recommended behaviour: Infants with bronchiolitis should not have a CXR, receive antibiotics, salbutamol, steroids or adrenaline. |
| --- |

Understanding the nature of the behaviour (conducting an assessment, reviewing the results and making a plan for care and disposition)

In your area is there a bronchiolitis guideline to refer to? Where would you find this / is it easy to use / do you agree with the content? How often would you refer to this or is it something that you know?

When a new colleague joins your team, how do they learn how bronchiolitis is managed in your department?

Do you have a role in assisting them with this? If so, what is your role?

*The following table lists prompts that may be used to explore the different barriers and facilitators to the behaviour, grouped according to the theoretical domains framework. The specific questions asked and the domains explored, will depend on the what is being discussed as well as the flow of the interview.*

**Draft prompt questions to explore factors influencing use of CXR:**

- Are there situations when you would order a CXR? Tell me about this situation?
- How many do you think receive one? i.e. percentage
- Do you see your colleagues ordering CXR? If so, why do you think they are doing this? Eg. Concerned re missing something, pressure from seniors / colleagues / caregivers

| **TDF Domains** | **Prompt Questions** |
| --- | --- |
| Knowledge | Are you colleagues/junior staff familiar with assessing infants with bronchiolitis? |
| Skills | Confidence in assessing bronchiolitis?  What skills / experience are needed?  Do you or your colleagues/junior staff find it difficult to interpret CXR findings to distinguish between that and pneumonia? |
| Social professional role and identity | Do you think ordering / questioning use of CXR is part of your role?  What happens if you question why a CXR is ordered?  Do you think doing / not doing a CXR is compatible or in conflict with current evidence? |
| Beliefs about capabilities | Do you feel confident in being able to determine the need for a CXR? What about your junior colleagues?  Are there any particular challenges in assessing bronchiolitis vs other resp illnesses and what tests if any are required?  Challenges in negotiating with families / radiology / colleagues?  What would help you in not feeling like you need to do a CXR? |
| Optimism | Do you feel that doing a CXR improves outcomes or your decision making processes? |
| Beliefs about consequences | Benefits / disadvantages of doing CXR.  Consequences of doing or not doing?  Any concern re medico-legal issues?  What is said / not said by colleagues if you do a CXR?  Do you think the evidence is strong enough to not do a CXR? |
| Reinforcements | Incentives / disincentives to do CXR? |
| Intentions | Do you consciously think about whether or not to CXR? |
| Goals | Why do CXRs get done?  Feel you have to or shouldn’t do?  Challenge to discharge without a CXR.  Does the guideline recommendations conflict with others? |
| Memory, attention and decision processes | Is ordering a CXR something you do routinely?  Will you need to make a concerted effort to not CXR? Do you think this is the same for others or different? |
| Environmental context and resources | Do resources influence whether you do a CXR?  Are radiology services available 24/7?  Is there any reminder / approval system when you order a CXR in this situation?  Are there any pressures in the ED / inpatient area to order a CXR? i.e. when planning to send infant home. |
| Social influences | Do you seek opinion of colleagues before doing a CXR or when reviewing it?  Views of colleagues?  Can you observe others eg have role models?  Are there any social influences / pressures that affect whether you perform a CXR (prompts, peers, families)? i.e when referring for admission |
| Emotion | Are there times when it is difficult to know whether to CXR?  Pressure from staff / families? |
| Behavioural regulation | Are there any guidelines to guide your practise?  What do you think would help you / your colleagues / environment change practice in regards to use of CXR? |

**Draft prompt questions to explore factors influencing use of salbutamol:**

- When managing an infant with bronchiolitis, are there situations where you would use salbutamol? Describe these.
- Do you see your colleagues prescribing salbutamol? If so, why do you think they are doing this? Eg. Trialling effect, family history of atopy / wheeze. Pressure / influence from seniors/ colleagues / caregivers.

| **TDF Domains** | **Prompt Questions** |
| --- | --- |
| Knowledge | What would make you give salbutamol?  Do you think giving / not giving salbutamol is compatible or in conflict with current evidence? |
| Skills | Know how to assess bronchiolitis and risk factors?  What skills / experience is needed? |
| Social professional role and identity | Do you think ordering / giving / questioning use of salbutamol is part of your role?  What happens if you question why salbutamol is prescribed?  Who can you discuss this with in your area?  Is there any time that you feel pressured to give salbutamol? |
| Beliefs about capabilities | Challenges in assessing bronchiolitis vs other resp illnesses and management / intervention that may be required?  Do you think your colleagues have difficulties in determining the need for salbutamol?  Challenges in negotiating with families / colleagues who may want to try salbutamol? How do you manage this? |
| Optimism | Do you feel that giving / trialing salbutamol improves outcomes / reduces LOS or alters your decision making processes? |
| Beliefs about consequences | Benefits / disadvantages of using salbutamol.  Consequences of doing or not doing?  What is said / not said by colleagues if you give salbutamol?  Any concern re medico-legal issues? |
| Reinforcements | Incentives / disincentives to give / trial salbutamol? |
| Intentions | Do you consciously think about whether or not to give salbutamol? |
| Goals | Why do you think salbutamol is give so often?  Feel you have to give or shouldn’t give?  Challenge to discharge without a treatment. |
| Memory, attention and decision processes | Is this something you do routinely? |
| Environmental context and resources | Do resources influence whether you trial salbutamol?  Are there any reminder systems around when to give or not give salbutamol?  Stressful nature of ED and pressure of sending infants home?  4 or 6 hour ED target? Availability of inpatient beds? |
| Social influences | Do you seek opinion of colleagues before giving salbutamol?  Views of colleagues?  Can you observe others eg have role models?  Are there any social influences that affect whether you give salbutamol (prompts, peers, families)? |
| Emotion | Are there times when it is difficult to know whether to trial salbutamol?  Pressure from staff / families to give or not give? How does that make you feel? |
| Behavioural regulation | Are there any guidelines to guide your practise?  What do you think would help you / your colleagues / environment change practice in regards to use of salbutamol? |

**Draft prompt questions to explore factors influencing use of glucocorticoids:**

- When managing an infant with bronchiolitis, are there situations where you would prescribe glucocorticoids? Describe these.
- Do you see your colleagues prescribing glucocorticoids? If so, why do you think they are doing this? Eg. Believe they are effective, family / past history of atopy / wheeze. Pressure / influence from seniors / colleagues / caregivers.

| **TDF Domains** | **Prompt Questions** |
| --- | --- |
| Knowledge | Do you think giving / not giving glucocorticoids is compatible or in conflict with current evidence?  What would make you prescribe glucocorticoids? |
| Skills | Confident in assessing bronchiolitis and risk factors?  Do you think your colleagues have the skills/experience to determine the need for glucocorticoids?  What skills / experience is needed? |
| Social professional role and identity | Do you think ordering / giving / questioning use of glucocorticoids is part of your role?  What happens if you question why it has been prescribed?  Who can you discuss this with in your area?  Is there any time that you feel pressured to give glucocorticoids? Describe. What would have helped? |
| Beliefs about capabilities | Challenges in assessing bronchiolitis vs other resp illnesses and management / intervention that may be required? Eg. asthma  Challenges in negotiating with families / colleagues who may advocate using glucocorticoids? How do you manage this?  Any concern re medico-legal issues? |
| Optimism | Do you feel that giving glucocorticoids improves outcomes / reduces LOS or alters your decision making processes? |
| Beliefs about consequences | Benefits / disadvantages of using glucocorticoids.  Consequences of doing or not doing?  What is said / not said by colleagues if you give them?  Any concern re medico-legal issues? |
| Reinforcements | Incentives / disincentives to give glucocorticoids? |
| Intentions | Do you consciously think about whether or not to give glucocorticoids? |
| Goals | Why do you think glucocorticoids are given so often?  Feel you have to give or shouldn’t give?  Challenge to discharge without treatment. |
| Memory, attention and decision processes | Is this something you do routinely? |
| Environmental context and resources | Do resources influence whether you give glucocorticoids?  Are there any reminder systems around when to give or not give?  Stressful nature of ED and pressure to send infants home?  4 or 6 hour ED target? Availability of inpatient beds? |
| Social influences | Do you seek opinion of colleagues before giving glucocorticoids? Who?  What are the views of your colleagues?  Can you observe others eg have role models?  What happens if they have been prescribed in the community? Do you stop the course?  Are there any social influences that affect whether you give glucocorticoids (prompts, peers, families)? |
| Emotion | Are there times when it is difficult to know whether to give glucocorticoids?  Pressure from staff / families to give or not give? How does that make you feel? |
| Behavioural regulation | Are there any guidelines to guide your practise?  What do you think would help you / your colleagues / environment change practice in regards to use of glucocorticoids? |

**Draft prompt questions to explore factors influencing use of adrenaline:**

- When managing an infant with bronchiolitis, are there situations where you would prescribe adrenaline? Describe these.
- Do you see your colleagues prescribing adrenaline? If so, why do you think they are doing this? Eg. Believe they are effective, family / past history of severe bronchiolitis. Pressure / influence from seniors / colleagues / caregivers.

| **TDF Domains** | **Prompt Questions** |
| --- | --- |
| Knowledge | What would make you prescribe adrenaline?  Do you think giving / not giving adrenaline is compatible or in conflict with current evidence? |
| Skills | Do you think your colleagues know how to assess bronchiolitis and risk factors?  Do you think your colleagues have the skills/experience to determine the need for adrenaline? |
| Social professional role and identity | Do you think ordering / giving / questioning use of adrenaline is part of your role?  What happens if you question why it has been prescribed?  Who can you discuss this with in your area?  Is there any time that you feel pressured to give adrenaline? Describe. What would have helped? |
| Beliefs about capabilities | Challenges in assessing bronchiolitis vs other resp illnesses and management / intervention that may be required? Eg. croup  Challenges in negotiating with families / colleagues who may advocate use of adrenaline? How do you manage this? |
| Optimism | Do you feel that giving adrenaline improves outcomes / reduces LOS or alters your decision making processes? |
| Beliefs about consequences | Benefits / disadvantages of using adrenaline.  Consequences of doing or not doing?  What is said / not said by colleagues if you give adrenaline?  Any concern re medico-legal issues? |
| Reinforcements | Incentives / disincentives to give adrenaline? |
| Intentions | Do you consciously think about whether or not to give adrenaline? |
| Goals | Why do you think adrenaline is given ?  Feel you have to give or shouldn’t give?  Challenge to discharge without having given a treatment.  Do you feel pressured to have tried everything before admitting? |
| Memory, attention and decision processes | Is this something you do routinely? |
| Environmental context and resources | Do resources influence whether you give adrenaline? Eg. Need monitored bed space, longer time in ED or admission  Are there any reminder systems around when to give or not give?  Stressful nature of ED and pressure to send infants home?  4 or 6 hour ED target? Availability of inpatient beds? |
| Social influences | Do you seek opinion of colleagues before giving adrenaline? Who?  What are the views of your colleagues?  Can you observe others eg have role models?  Are there any social influences that affect whether you give adrenaline (prompts, peers, families)? |
| Emotion | Are there times when it is difficult to know whether to give adrenaline?  Pressure from staff / families to give or not give? How does that make you feel? |
| Behavioural regulation | Are there any guidelines to guide your practise?  What do you think would help you / your colleagues / environment change practice in regards to use of adrenaline? |

**Draft prompt questions to explore factors influencing use of antibiotics:**

- When managing an infant with bronchiolitis, are there situations where you would prescribe antibiotics? Describe these.
- What would you do if an infant was already on antibiotics?
- Do you see your colleagues prescribing antibiotics? If so, why do you think they are doing this? Eg. Believe they are effective, past history of severe bronchiolitis. Pressure / influence from seniors / colleagues / caregivers. Infant already on them and not wanting to stop part way through course.

| **TDF Domains** | **Prompt Questions** |
| --- | --- |
| Knowledge | What would make you prescribe antibiotics?  What factors influence colleagues prescribing antibiotics?  Do you think giving / not giving antibiotics is compatible or in conflict with current evidence? |
| Skills | Know how to assess bronchiolitis and risk factors?  What skills / experience is needed to distinguish bronchiolitis from a bacterial LRTI? |
| Social professional role and identity | Do you think ordering / giving / questioning use of antibiotics is part of your role?  What happens if you question why it has been prescribed?  Who can you discuss this with in your area?  Is there any time that you feel pressured to give antibiotics? Describe. What would have helped? |
| Beliefs about capabilities | Challenges in assessing bronchiolitis vs other resp illnesses and management / intervention that may be required? Eg. Pneumonia.  Challenges in negotiating with families / colleagues who may want antibiotics? How do you manage this? |
| Optimism | Do you feel that giving antibiotics improves outcomes / reduces LOS or alters your decision making processes? |
| Beliefs about consequences | Benefits / disadvantages of using antibiotics.  Consequences of doing or not doing?  Any concern re medico-legal issues?  What is said / not said by colleagues if you give antibiotics? |
| Reinforcements | Incentives / disincentives to give antibiotics? |
| Intentions | Do you consciously think about whether or not to give antibiotics? |
| Goals | Why do you think antibiotics are given ?  Feel you have to give or shouldn’t give?  Challenge to discharge without them?  Would it ever impact on whether a child was admitted or not? |
| Memory, attention and decision processes | Is this something you do routinely? |
| Environmental context and resources | Do resources influence whether you give antibiotics? Eg. Family’s ability to get them / give them.  Are there any reminder systems around when to give or not give?  Stressful nature of ED and pressure to send infants home?  Attempt to reduce re-presentation? |
| Social influences | Do you seek opinion of colleagues before giving antibiotics? Who?  What are the views of your colleagues?  Can you observe others eg have role models?  Are there any social influences that affect whether you give antibiotics (prompts, peers, families)? |
| Emotion | Are there times when it is difficult to know whether to give antibiotics? Is there someone you can discuss this with?  Pressure from staff / families to give or not give? How does that make you feel? |
| Behavioural regulation | Are there any guidelines to guide your practise?  What do you think would help you / your colleagues / environment change practice in regards to use of antibiotics? |

**Realising changes in the ED / inpatient setting (ask at all interviews):**

How do practice changes generally get implemented?

What do you see as the perceived barriers to changing practice?

Are clinical guidelines routinely implemented or used in your department? How is this done? How do you keep up to date?

*Prompts: quality plan / group, change agent/champion? Clinical leadership? How are ‘topics’ prioritized? Do staff members generally get involved in how to change practices? Do you feel free to suggest ideas?*

What do you think is the best way to implement changes?

*Prompts: e.g., Education sessions? Reminder systems? Champion/opinion leaders? Audit and feedback? Posters? Other?*

**If there was one thing you could change in your hospital to improve the management of bronchiolitis, what would you change?**

**Other**

Is there anything else about the management of infants with bronchiolitis that you would like to mention that has not already been covered?

THANK YOU VERY MUCH FOR YOUR TIME

**Interview schedule for Emergency Department / General Paediatric Clinical Director (or delegate)**
**Introduction**
As outlined in the explanatory statement, this study aims to identify and explore the barriers and facilitators that influence management of infants with bronchiolitis. For the purpose of this interview, I am interested in infants <1 year of age with a diagnosis of bronchiolitis.

**You do not have to answer every question and can cease the interview at any time. If you need to attend to an urgent matter, we can stop the interview and recommence it later.**

You have signed a consent form previously. Can you confirm that you are still happy to proceed with the interview?

In addition to talking to clinical staff regarding the management of infants with bronchiolitis, we are also very interested to learn about the complex contextual and organisational environment of the ED / inpatient areas that may influence management.

**Before I start do you have any questions you would like to ask me?**

First, I would like to get an idea about the human resources that are available for the management of infants with bronchiolitis in your area? Eg Which professions would be involved, and what would be the number of staff for each profession and the number FTE’s for each of them?

What do you think about the availability of resources for managing patients with bronchiolitis in this ED / inpatient area?

Eg availability of team members during out of hours?

What type(s) of medical record system is in use? Paper based / electronic?

Use of reminders/prompts/pathways in system?

Can you tell me about the work environment in the ED / inpatient area in relation to quality improvement / change management? [*organisational culture*]

What would you tell new staff about how ‘things are’ in the ED / inpatient area?

What are the unwritten rules that relate to changing practice in the ED / inpatient area?

How do changes generally get implemented?

*Prompts: change agent/champion? Clinical leadership? How are ‘topics’ prioritized?*

Has your department been involved in quality improvement projects in the past, either internally or externally initiated?

Can you briefly describe some examples?

What turned out to be the key lessons from these experiences?

What do you see as the perceived barriers to changing practice?

What do you see as the perceived enablers to changing practice?

Can you tell me about the use of guidelines / protocols / organizational policies in general and for the management of bronchiolitis in your area?

What do you think about the use of these tools in the ED / inpatient setting?

What, if any, protocols and/or guidelines are in use? How are they accessed by staff?

Are you aware of any guidelines / protocols used for the management of infants with bronchiolitis? If so, are they externally / internally developed?

Who decides upon the exact work processes in the ED / inpatient area? Would staff generally be involved?

Who decides upon what tools or methods to use for, say, management of bronchiolitis?

What about the communication between ED / inpatient staff? What channels are used, and do you think these work well?

*Prompts: medical record, face-to-face, phone, pager (disruptive nature)*?

What processes (if any) are in place for feedback?

*Prompts: feedback on routinely collected data? (Near)-mistakes? Individual/team/departmental level?*

Often, a strong incentive for making changes is when things go (almost) wrong. What systems, if any, are in place to learn from mistakes?

*Prompts: (blame-free) reporting systems? Discussion of events with people from other ward / organization? Would underlying systems / work processes be questioned / redesigned if needed? [‘double loop’ versus ‘single loop’ learning]*

Are there organizational or legal regulations that influence how things are done in the ED / inpatient area in general? And for the management of bronchiolitis specifically?

*Prompts: eg ‘4/6 hour rule’’🡪 how is that translated into the work processes / does it influence work processes and –if so- how? Any medico-legal issues?*

What would be the incentives for you / your department to participate in a KT/QI project on the management of bronchiolitis / in general?

Any other matters that you think may be relevant to influence / limit / stimulate EB care?

NB: If the interviewee has clinical responsibility of infants with bronchiolitis, questions from previous schedule around management of infants with bronchiolitis will also be asked. Is there an aspect of the patient pathway we should pay more attention to in future interviews?

If there was one thing you could change in your hospital to improve the management of bronchiolitis in ED / inpatient area what would you change?

Any other matters that may be relevant in managing patients with bronchiolitis that has not been mentioned yet?

**THANK YOU VERY MUCH FOR YOUR TIME**
