# Supplementary material for: Understanding factors that contribute to variations in bronchiolitis management in acute care settings: a qualitative study in Australia and New Zealand using the Theoretical Domains Framework
Source: BMC Pediatr. 2020 May 1;20:189. doi: 10.1186/s12887-020-02092-y (PMC7193400; doi:10.1186/s12887-020-02092-y)
Supplement: Supplementary file 3 — Additional file 3 Table S1. Factors thought to influence practice in caring for infants with bronchiolitis in regard to chest x-ray. Table S2. Factors thought to influence practice in caring for infants with bronchiolitis in regard to salbutamol use. Table S3. Factors thought to influence practice in caring for infants with bronchiolitis in regard to antibiotic use. Table S4. Factors thought to influence practice in caring for infants with bronchiolitis in regard to glucocorticoid use. Table S5. Factors thought to influence practice in caring for infants with bronchiolitis in general. [file 12887_2020_2092_MOESM3_ESM.docx]

**Additional file 3 (Table S1 to S5):**

Table S1: Factors thought to influence practice in caring for infants with bronchiolitis in regard to chest x-ray

| **Key Domain** | **Themes** | **Dr** | **Nurse** | **Representative quotations** |
| --- | --- | --- | --- | --- |
| **Beliefs about consequences** | Aware of risks of radiation and long term risks of cancer; only small amount of radiation.  Concern at level of respiratory distress, deterioration, missing something, CXR may confirm diagnosis. | ✓  ✓  ✓  ✓  ✓  ✓  ✓ |  | *What concerns you is if the child has been in eight times for bronchiolitis and they’ve had six x-rays, and you, kind of, think that’s getting unnecessary.* (Senior Medical Officer (SMO), Paediatric Inpatients, Regional)  *They’re just doing extra things that are unnecessary and costly and maybe just have a little bit of morbidity in the sense you’ve given them a small amount of radiation.*  (SMO, Paediatric Inpatients, Metropolitan)  *There's no doubt our junior staff are aware of the risks of ionising radiation and long term risks of cancer.*  (SMO, Paediatric Inpatients, Regional)  *It’s not as though people are failing to do something that’s going to cause morbidity or mortality particularly. They’re just doing extra things that are unnecessary and costly and maybe just have a little bit of morbidity in the sense you’ve given them a small amount of radiation.*  (SMO, Paediatric Inpatients, Metropolitan)  *I guess the one area for our juniors would be their concern of are they missing something else, so the bronch that they end up x-raying at 3:00 in the morning because, you know, it’s a classical thing, they end up because they’re not really sure.*  (SMO, Paediatric ED, Metropolitan)  *I think they’re worried about deterioration. And I think worried about whether a child should be on IV antibiotics.*  (SMO, Paediatric ED, Metropolitan)  *Yeah, I think, I mean it’s partly like, oh, okay, you’re not responding, you’re going downhill, you’re kind of… So I’d get an X-ray, I’d do a gas, I’d do a, so that, it’s almost a reassessment to make sure that this is indeed the diagnosis.*  (SMO, Paediatric Inpatients, Metropolitan) |
| **Knowledge** | Lack of knowledge and experience in caring for infants with bronchiolitis. | ✓ | ✓ | *It’s probably a bit of lack of knowledge and lack of experience; you’ve not seen enough, you’re just worried. They can look quite bad and you are in a position where you don’t really want to miss something, although you’ve really got nothing to miss.*  (Resident Medical Officer (RMO), Paediatric ED, Metropolitan)  *Sometimes they might do an x-ray. I think that’s just more a knowledge deficit than anything else*.  (Senior nurse, Paediatric Inpatients, Metropolitan) |
| **Skills** | Lack of confidence / competence in caring for infants with bronchiolitis. | ✓ |  | *Perhaps they’re just not that confident in their clinical skills or not that comfortable with bronchiolitis where the kids often look very unwell in terms of respiratory distress.*  (SMO, Paediatric Inpatients, Metropolitan) |
| **Social influences** | Parental expectations.  Pressure from staff members. | ✓  ✓  ✓  ✓ | ✓ | *So there are parents that come in with expectations.*  (RMO, Paediatric Inpatients, Metropolitan)  *So I think there is some parental reassurance that goes on as well.*  (SMO, Paediatric ED, Metropolitan)  *Well pressure comes from parents.*  (SMO, Paediatric ED, Metropolitan)  *I think they are worried about missing something, so there’s certainly no pressure from nursing staff, so I think that they’re worried that when it comes to a senior doctor reviewing the patient that they’ll be like, “Well, have you done a chest x-ray?” or something like that, so I think they’re worried that they will miss something.*  (RN, Paediatric Inpatients, Metropolitan)  *And if the child is still in ED, then you may be asked to get a CXR and you may be asked to maybe trial salbutamol in possibly the older ones, more than 6 months.*  (RMO, Paediatric ED, Metropolitan) |
| **Environmental context and resources** | Junior medical staff with reduced senior support after hours.  Regional hospitals – retrievals, distance to tertiary care  Challenges of staff turnover, non-paediatric staff, overseas trained doctors. | ✓  ✓  ✓ | ✓ | *A brand-new Senior House Officer who has only seen a handful of bronchiolitis, who’s on overnight, who’s got a baby who’s working [hard] and has a fever, then I think that they will be more inclined to do an x-ray.*  (SMO, Paediatric Inpatients, Regional)  *I suppose from a retrieval’s perspective, you really want to make sure it’s bronch and nothing else. And you’re not going to get a surprise on route as well.*  (SMO, Paediatric Inpatients, Metropolitan)  *Once we’ve had them in the department for a little while they’d start to learn that we aren’t, we don’t practise like that and we do try and reserve x-rays and give the spiels about radiation and things.*  (Senior RN, Paediatric ED, Metropolitan)  *And I suppose the more regional you go the more likely that you are to have overseas trained doctors as the emergency department staff, which probably also contributes to it.*  (SMO, Paediatric Inpatients, Metropolitan) |

Table S2

Factors thought to influence practice in caring for infants with bronchiolitis in regard to salbutamol use

| **Key Domain** | **Themes** | **Dr** | **Nurse** | **Representative quotations** |
| --- | --- | --- | --- | --- |
| **Beliefs about consequences** | Clinicians differing view on benefit and potential harm in using salbutamol in.  . | ✓  ✓    ✓  ✓  ✓  ✓ |  | *If someone can be made better and go home, then it’s worth trying.*  (SMO, Paediatric ED, Metropolitan)  *You’re not going to necessarily run into harm by giving it a try.*  (SMO, Paediatric Inpatients, Metropolitan)  *There’s always going to be difference in practice and I think that you can’t really write a rule for those six to 12 monthers because those of us that have always used it intermittently for those 10, 11 monthers that you know anecdotally it works for, we’re probably not going to change practice significantly.*  (SMO, Mixed adult/paediatric ED, Regional)  *So a child who’s been recurrently wheezy, or recurrent episodes of bronchiolitis, and they’re 10 or 11 months, then I think you’ll get variation in practice, because I think some clinicians would trial Salbutamol. I think the closer you are to zero, the less variability there is.*  (SMO, Paediatric ED, Metropolitan)  *There's no doubt that we see nine month olds who are covered in eczema with a bad family history of asthma who are moderately severe. Bronchiolitis that - you know, I give them a Ventolin nebuliser, a spacer - especially if Mum says, 'Look, that's helped in the past and his brother's got asthma,' and this kid's covered in eczema. As opposed to if it's a three month old, we'd probably never give them a trial.*  (SMO, Paediatric Inpatients, Regional)  *I would still trial it in a 10, 11 monther who’s atopic, with the understanding that an explanation to parents and to the junior who’s seen them that this isn’t going to change duration, this isn’t going to change how long they are sick for, but if it means they feed a bit better and they sleep a bit better that child may be able to go home rather than be admitted, or whatever it may be. So that would be my practice around that in that under 12 monther but getting close to 12 months, and I’ve always done that.*  (SMO, Mixed adult/paediatric ED, Regional) |
| **Knowledge** | Deficit in knowledge (senior and junior) re evidence of salbutamol use. | ✓  ✓ | ✓ | *Because most of us don’t at a more senior level [give salbutamol], if we see a deteriorating bronchiolitis, we’re more willing to accept that as progression of the underlying condition, as opposed to lack of treatment with salbutamol.*  (SMO, Paediatric Inpatients, Metropolitan)  *If you are going to try a bronchodilator, why are you doing this? And what’s the evidence behind it and understanding the lack of evidence behind it, and doing that in conjunction with a senior clinician.*  (SMO, Mixed adult/paediatric ED, Regional)  *I think they are, kind of mixing the asthma / viral induced wheeze with the bronchiolitis one; so we were going by our experience with the patient whereas I feel like sometimes they are drawing on experience with something else.*  (Registered Nurse (RN), Paediatric Inpatients, Metropolitan) |
| **Social/professional role and identity** | Nurses guiding junior doctors in the care of infants with bronchiolitis.  Nurses’ not feeling expertise is listened to. | ✓ | ✓  ✓ | *And in fact, our nurses are one of the most influential group. Because they question the juniors; the junior doctors.*  (SMO, Paediatric ED, Metropolitan)  *There is the occasion where you may get somebody that wants to trial some salbutamol on a child who is less than 1 and that may or may not have atopy and then it’s going to be the nurse, as her role to query that in my experience.*  (RN, Paediatric ED, Metropolitan)  *There are so many children who are charted salbutamol where the nursing staff are saying, “Look, this child isn’t responsive” but then one of the medical team will go and hear something completely different from what the nurses hear, so they’ll say, “Yes, they are partially responsive. You should continue.” So, nursing staff will continue, but still not think it’s effective.*  (RN, Paediatric Inpatients, Metropolitan) |
| **Social influences** | Some degree of parental and clinician pressure to trial salbutamol. | ✓  ✓  ✓ |  | *I think the commonest reason for its use, well, the two things is people wanting to give them something, so parental pressure and clinicians’ internal pressure to give some kind of treatment for an illness that there is actually no additional treatment for and misunderstanding about the idea that it’s not actually salbutamol responsive wheeze.*  (SMO, Mixed adult/paediatric ED, Regional)    *So they often asked, “Have you tried salbutamol?” (when discussing referring for inpatient care)*  (RMO, Mixed adult/paediatric ED, Regional)  *Because you do get parents coming in and saying, “actually they were like this, six weeks ago. The G.P. gave me a blue inhaler, and it worked.”*  (SMO, Paediatric ED, Metropolitan) |

Table S3

Factors thought to influence practice in caring for infants with bronchiolitis in regard to antibiotic use

| **Key Domain** | **Themes** | **Dr** | **Nurse** | **Representative quotations** |
| --- | --- | --- | --- | --- |
| **Beliefs about consequences** | Mixed opinions on risks of antibiotic use.  Concern re deprived populations and treatment of early bronchiectasis. | ✓  ✓  ✓ |  | *48 hours of antibiotics in terms of the big picture of antibiotic resistance isn’t going to make that much of a difference.*  (SMO, Paediatric ED, Metropolitan)  *So if you say that, you know, there’s no value, stop the antibiotics and you’ve got a Mum saying, well actually they had a bit of a fever last night and, you know, they’re working hard and, and there’s also a belief that antibiotics are not harmful. So it’s like, well I’m not going to do any harm, they’ve already taken two or three days (speaking about junior colleagues).*  (SMO, Paediatric ED, Metropolitan)  *We do have quite a high antibiotic prescribing rate but we think that we’ve also got a high bronchiectasis problem. So being too strict about it being just viral, I might leave our high Māori deprivation, smoke exposed kids to not being appropriately treated for early bronchiectasis.*  (SMO, Paediatric Inpatients, Regional) |
| **Social influences** | Parental expectations to receive antibiotics. | ✓ | ✓ | *I think they do [in regard to doctors having a concern about the consequences of giving antibiotics] but they are focusing on the short term as opposed to the long term, so in the short term they’re keeping the family happy.*  (RN, Paediatric Inpatients, Metropolitan)  *So there are parents that come in with expectations.*  (RMO, Paediatric Inpatients, Metropolitan) |
| **Knowledge** | Lack of knowledge and experience in caring for infants with bronchiolitis. |  | ✓ | *Which is why so many children get treated with antibiotics, I think. There’s concern that there’s not quite enough wheeze. That leads to an x-ray. There’s abnormalities on the x-ray. That leads to treatment with antibiotics.*  (Senior RN, Paediatric Inpatients, Metropolitan) |

Table S4

Factors thought to influence practice in caring for infants with bronchiolitis in regard to glucocorticoid use

| **Key Domain** | **Themes** | **Dr** | **Nurse** | **Representative quotations** |
| --- | --- | --- | --- | --- |
| **Knowledge** | Variation in knowledge of glucocorticoid use in infants with bronchiolitis | ✓  ✓  ✓ | ✓ | *I was very surprised by the high use of it in the community, you know, often the families were coming and not only having had steroid but have a stash of steroid at home and are using that.*  (SMO, Paediatric Inpatients, Regional)  *I think the juniors here are pretty confident that there’s no place for steroids in bronchiolitis.*  (SMO, Paediatric ED, Metropolitan)  *I think treatment is probably more than recognition, because the risk is that, of them thinking (junior medical staff), “Well, I gave that two-and-a-half-year-old Redipred last week, now I’m going to give this baby, because of the wheeze he’s got”.*  (SMO, Mixed adult/paediatric ED, Regional)  *We’re very good at using steroids a lot, a lot of steroids. [Laughing] In fact most kids that come in with any kind of wheeze like that will get steroids. So probably that sort of thing would be assessing on what kind of, if they’re a mild bronchiolitis do we really need steroids? Is it their third presentation in a couple of weeks? Is it now time to use steroids?*  (RN, Mixed adult/paediatric ED, Regional) |
| **Beliefs about consequences** | Potential harmful effects of glucocorticoids / comfortable to stop glucocorticoids | ✓  ✓  ✓  ✓ |  | *So on a ‘primum non nocere’ principle that steroids could actually predispose to immune suppression, I saw one fatal pneumonia from recurrent courses of Redipred prescribed to a baby that had barely reached term, or not long past it.*  (SMO, Paediatric Inpatients, Regional)  *I’m very keen on stopping steroids. And a very, I mean I’ll just have the conversation that they don’t, they’re not, you know, there’s no evidence and they’re not beneficial. In fact, we don’t like giving, you know, steroids recurrently to children.*  (SMO, Paediatric ED, Metropolitan)  *If they are diagnosed with bronch, we generally wouldn’t continue oral steroids or anything like that.*  (SMO, Paediatric Inpatients, Metropolitan)  *I thinking pretty comfortable, so steroids especially (to stop a course). I would stop it and cross it off, yeah.*  (SMO, Paediatric Inpatients, Metropolitan) |
| **Beliefs about capabilities** | Importance of maintaining good relationships with primary care providers. | ✓ |  | *You need families to trust their General Practitioners. So you don’t want to run them down and say, “This is ridiculous, stop it”.*  (RMO, Paediatric ED, Metropolitan) |
| **Social/professional role and identity** | Cognizant of strong relationships with primary health care providers.  Impact of senior staff on juniors practice | ✓ | ✓ | *You need families to trust their General Practitioners. So you don’t want to run them down and say, “This is ridiculous, stop it”.*  (RMO, Paediatric ED, Metropolitan)  *There is that kind of a little bit of a hierarchical structure sometimes still.*  (RN, Paediatric Inpatients, Regional) |
| **Environmental context and resources** |  | ✓ |  | *We’ve had quite a few American consultants come through who have quite different opinions, so it’s actually quite difficult for our juniors.*  (SMO, Mixed adult/paediatric ED, Regional) |
| **Social influences** | Parental pressure prescribing steroids | ✓ | ✓  ✓ | *Especially if you’ve got a kid with recurrent bronchiolitis the parents will go “This is what works. This is the only thing that’s worked in the past. We’ve tried this and this; we always have to go to steroids at the end of the day”.*  (RN, Mixed adult/paediatric ED, Regional)  *I always sort of think, would you give this to your own kids, would I take this myself?*  (SMO, Paediatric Inpatients, Regional)  *I think it’s a combined thing of pressure of parents, and also the junior docs kind of like “Well we want to be seen to be doing something. We don’t want to give, send you away with not giving you anything. We’re not going to give you antibiotics, but just do something.*  (RN, Mixed adult/paediatric ED, Regional) |

Table S5

Factors thought to influence practice in caring for infants with bronchiolitis in general

| **Key Domain** | **Themes** | **Dr** | **Nurse** | **Representative quotations** |
| --- | --- | --- | --- | --- |
| **Beliefs about consequences** | Concern re deterioration and missing something  or wanting to prevent deterioration by doing something | ✓  ✓ | ✓ | *This kid is looking quite sick, I should be doing something.*  (SMO, Paediatric ED, Metropolitan)  *I guess people just feel they want to do something for it, if that makes sense, so they think prescribing medications and things is going to, they just kind of hit them with everything that they think might work, rather than what’s actually in the guidelines.*  (Senior RN, Mixed adult/paediatric ED, Metropolitan)  *I think they’re worried about deterioration.*  (SMO, Paediatric ED, Metropolitan) |
| **Knowledge** | Positive response to Australasian bronchiolitis guideline.  Lack of knowledge of bronchiolitis in junior clinicians. | ✓  ✓  ✓ | ✓ | *Once you have very clear protocol type guideline as to what to do and when to do it, then it gives people a recipe to follow and they’re more likely to follow it.*  (SMO, Paediatric Inpatients, Metropolitan)  *It’s probably a bit of lack of knowledge and lack of experience; you’ve not seen enough, you’re just worried. They can look quite bad and you are in a position where you don’t really want to miss something, although you’ve really got nothing to miss.*  (RMO, Paediatric ED, Metropolitan)  *Yeah, I think it’s lack of knowledge and seniority, really. The more junior you are, you, I guess, tend to do more things, because you think it’s the right thing to do and it might not be the right thing to do.*  (Senior Registered Nurse, Paediatric ED, Metropolitan)  It’s hard to look at a bronch baby for your first time and know what to do.  (SMO, Paediatric Inpatients, Regional) |
| **Social/professional role and identity** | Importance of good relationships between clinicians | ✓  ✓ |  | *I think junior doctors are much more confident in this generation than maybe in the previous generation just to ring up their boss and say ‘Look, I just want to chat to you about a patient’.*  (SMO, Paediatric Inpatients, Regional)  *And in fact, our nurses are one of the most influential group. Because they question the juniors, the junior doctors.*  SMO, Paediatric ED, Metropolitan. |
| **Environmental context and resources** | Regional / access issues – staffing and skill mix in mixed adult/paediatric EDs.  Time pressures to make decisions leading to interventions.  Challenges with turnover of staff  Increasing use of high-flow oxygen. | ✓  ✓  ✓  ✓  ✓  ✓ | ✓  ✓ | *It’s terrifying! (in response to “it must be quite stressful at night?”)*  (RMO, Mixed adult/paediatric ED, Regional)  *One of the things in our ED is usually they’re not a paediatric nurse. So they’re often adult nurses, so often they may have a different view.*  (SMO, Mixed adult/paediatric ED, Regional)  *You can see why they do plunge in for x-ray straight away ‘cause that’s happened in an adult which is, where a lot of these trainees are coming from is just seeing adults and haven’t really actually seen a lot of these little ones.*  (SMO, Paediatric Inpatients, Regional)  *When it comes to even orientation and stuff the paeds stuff takes a very big back seat to everything else.*  (RN, Mixed adult/paediatric ED, Regional)  *Perhaps, maybe the time pressure of needing to make an assessment; is this bronch or is it something else?*  (SMO, Paediatric Inpatients, Regional)  *I guess you just kind of capture them, everyone gets up to speed, and then you’ve got a whole new bunch that will come through.*  (Senior RN, Mixed adult/paediatric ED, Regional)  *And of course high-flow has been a game changer, so the problem is we're probably now overusing that. That's probably better than giving them steroids and things they don't need.*  (SMO, Paediatric Inpatients, Regional)  *Machines that go beep, and it looks impressive to the family, and we have a real perception that it's one of those.*  (SMO, Paediatric Inpatients, Regional) |
| **Skills** | Role modelling of interactions with families in regards to support being provided.  Lack of confidence due to less experience of managing bronchiolitis. | ✓  ✓ | ✓ | *But I think we also under describe what we are doing. Like I think honouring nursing care at its best, we do that insufficiently at times. So for example, I think it should be phrased quite positively. We are maintaining fluids and all those regimens.*  (SMO, Paediatric Inpatients, Regional)  *It’s such a nursing sort of condition and nursing managed and nursing led case that I think is key to be working with those families and giving them a really good understanding.*  (Senior RN, Paediatric ED, Metropolitan)  *I think a lot of it is how comfortable ED physicians are, not doing anything. I guess in medicine, you feel somebody has come to you with a problem, you need to fix it. Whereas sometimes you just need to give time.*  (RMO, Paediatric ED, Metropolitan) |

ED: Emergency Department

RMO: Resident Medical Officer (Registrar, House Officer, Trainee)

RN: Registered Nurse

SMO: Senior Medical Officer (Consultant, Clinical Director)
